# Combined impact of prediabetes and hepatic steatosis on cardiometabolic outcomes in young adults

**DOI:** 10.1186/s12933-024-02516-4

**Published:** 2024-11-21

**Authors:** Wonsuk Choi, Minae Park, Sojeong Park, Ji Yong Park, A Ram Hong, Jee Hee Yoon, Kyoung Hwa Ha, Dae Jung Kim, Hee Kyung Kim, Ho-Cheol Kang

**Affiliations:** 1 Department of Internal Medicine, Chonnam National University Hwasun Hospital, Chonnam National University Medical School, 322, Seoyang-ro, Hwasun-eup, Hwasun-gun, Hwasun, 58128 Jeollanam-do Republic of Korea; 2 Department of Biological Chemistry, University of California Irvine School of Medicine, Irvine, CA USA; 3 Data Science Team, Hanmi Pharm. Co., Ltd, Seoul, Korea; 4 Department of Endocrinology and Metabolism, Ajou University School of Medicine, Suwon, Korea

**Keywords:** Prediabetes, Hepatic steatosis, Young adults, Cardiometabolic disease, Cohort analysis

## Abstract

**Objectives:**

This study aimed to investigate the impact of hepatic steatosis on cardiometabolic outcomes in young adults with prediabetes.

**Methods:**

A nationwide cohort study was conducted with 896,585 young adults under 40 years old without diabetes or previous history of cardiovascular disease. Hepatic steatosis was identified using a fatty liver index of ≥ 60. The outcomes of this study were incident diabetes (DM) and composite major adverse cardiovascular events (MACE), including myocardial infarction, stroke, or cardiovascular death.

**Results:**

During a median follow-up of 11.8 years, 27,437 (3.1%) incident DM cases and 6,584 (0.7%) MACE cases were recorded. Young adults with prediabetes had a significantly higher risk of incident DM (hazard ratio [HR]: 2.81; 95% confidence interval [CI]: 2.74–2.88; *P-*value: <0.001) and composite MACE risk (HR: 1.10; 95% CI: 1.03–1.17; *P-*value: 0.003) compared to individuals with normoglycemia, after adjusting for relevant covariates. Stratification based on hepatic steatosis showed that the combination of prediabetes and hepatic steatosis posed the highest risk for these outcomes, after adjusting for relevant covariates. For incident DM, the HRs (95% CI; *P*-value) were: 3.15 (3.05–3.26; <0.001) for prediabetes without hepatic steatosis, 2.89 (2.78–3.01; <0.001) for normoglycemia with hepatic steatosis, and 6.60 (6.33–6.87; <0.001) for prediabetes with hepatic steatosis. For composite MACE, the HRs (95% CI; *P*-value) were 1.05 (0.97–1.13; 0.235) for prediabetes without hepatic steatosis, 1.39 (1.27–1.51; <0.001) for normoglycemia with hepatic steatosis, and 1.60 (1.44–1.78; <0.001) for prediabetes with hepatic steatosis.

**Conclusions:**

Prediabetes and hepatic steatosis additively increased the risk of cardiometabolic outcomes in young adults. These findings hold significance for physicians as they provide insights into assessing high-risk individuals among young adults with prediabetes.

**Supplementary Information:**

The online version contains supplementary material available at 10.1186/s12933-024-02516-4.

## Introduction

Prediabetes refers to elevated glucose levels below the diabetes (DM) criteria, signaling improper carbohydrate metabolism [1]. Prediabetes is regarded as a risk factor for the progression to DM and cardiovascular diseases (CVD) rather than as a separate disease entity [2]. Previous studies show that prediabetes significantly increases CVD risk in global populations [3].

The prevalence of prediabetes is growing among young adults [4]. Previous studies have associated prediabetes with an increased CVD risk in this demographic [5, 6]. Healthcare providers need to maintain clinical vigilance on young adults starting from the prediabetes stage, given the significantly higher impact of early-onset type 2 DM on CVD than that of those with late-onset type 2 DM [7, 8] or type 1 DM [9]. Moreover, identifying high-risk individuals among young adults with prediabetes based on predictive clinical factors for the progression to diabetes and CVD is crucial. However, limited research exists on this issue.

Individuals with hepatic steatosis face an increased risk of T2DM and CVD [10–12]. Among high-risk individuals for T2DM, those with hepatic steatosis have the highest risk of developing type 2 DM and CVD [13]. However, the association between hepatic steatosis and the progression to diabetes and CVD in young adults with prediabetes remains uncertain.

Therefore, this study aimed to investigate the effect of hepatic steatosis, assessed through the fatty liver index (FLI), on cardiometabolic outcomes, including incident DM and major adverse cardiovascular events (MACE), in young adults with prediabetes using a Korean nationwide population cohort. The objective was to determine the impact of prediabetes and/or hepatic steatosis on the risk of incident diabetes and MACE in this demographic group.

## Methods

### Data source

The Korean National Health Insurance (NHIS) datasets covering claims and preventive health check-ups in Korea from January 2009 to December 2012 were employed in this study. The NHIS is a single payer healthcare system administered by the Korean government that insures more than 97% of the Korean population. This database includes medical diagnoses classified by International Classification of Diseases 10th revision (ICD-10) codes, alongside comprehensive details regarding prescriptions, procedures, hospital visits, and hospitalizations [14]. The health check-up database includes lifestyle and behavior questionnaires alongside anthropometric and laboratory measurements. Previous studies have detailed methods for these measurements [15, 16]. Additionally, nationwide death certificate data from the Korean National Statistical Office was employed.

## Study cohort

Individuals aged ≥ 20 years without DM (defined by fasting glucose levels of 126 mg/dL or higher, presence of ICD-10 codes E10-14, or a history of glucose-lowering medication before the index date) and who underwent routine health check-ups with FLI calculation data from January 2009 to December 2012, were selected. Out of 3,602,623 participants, 2,706,038 individuals were excluded due to age (≥ 40 years), cardiovascular (ICD-10 codes I21–22, I48, and I63–64) or liver disease excluding nonalcoholic fatty liver disease (NAFLD) (ICD-10 codes K75.1–75.4, K75.9, and K76.1–76.9), alcohol consumption ≥ 3 times per week, any cancer diagnosis (ICD-10 codes C00–97), rheumatic mitral valve disease (ICD-10 codes I05), and cardiac/vascular implants or grafts (ICD-10 code Z95), or missing data. Overall, 896,585 subjects were included in this study (Supplementary Fig. 1), with the index date defined as the first day FLI calculation data were collected.

## Patient and public involvement

Patients and the public were not involved in any aspect of this study.

## Defining prediabetes

Normoglycemia was defined as fasting glucose levels < 100 mg/dL in individuals without a history of diabetes, while prediabetes was defined as levels between 100 and 125 mg/dL in the same population during the baseline evaluation.

## Defining hepatic steatosis

Hepatic steatosis was defined as FLI ≥ 60 in the main analysis, using a widely accepted and validated non-invasive diagnostic test. In the sensitivity analysis, it was defined as FLI ≥ 30. This test has an area under the receiver operating characteristic curve of 0.844, with positive predictive values of 83.2% and 84.8% and negative predictive values of 65.3% and 87.4% for Asian males and females, respectively [17, 18]. FLI was calculated using the following equation: (exp(0.953 × log(triglycerides) + 0.139 × body mass index (BMI) + 0.718 × log(gamma-glutamyl transferase, GGT) + 0.053 × waist circumference − 15.745))/(1 + exp(0.953 × log(triglycerides) + 0.139 × BMI + 0.718 × log(gamma-glutamyl transferase) + 0.053 × waist circumference − 15.745)) × 100 [19].

### Study endpoint and follow-up

The outcomes of this study were incident DM and composite MACE, including myocardial infarction (MI), stroke, or cardiovascular death. Incident DM was defined as a new DM diagnosis (ICD-10 codes E10-14) accompanied by a prescription for glucose-lowering medications. MI was defined as hospitalization with ICD-10 codes I21 or I22. Stroke was defined as ICD-10 codes I63 or I64 during hospitalization with brain imaging claims such as magnetic resonance imaging or computed tomography. Cardiovascular death was defined using ICD-10 codes I00–99. The outcomes for the exploratory analysis were incident hepatocellular carcinoma (HCC) and liver-related death. HCC was defined as a diagnosis in the primary or secondary position in the inpatient setting using ICD-10 code C22. Liver-related death was defined using ICD-10 codes K00–K95. The study population was followed from the index date until the occurrence of each study outcome, death, or the end of the study period on December 31, 2021.

## Variable definitions

Information on current smoking, alcohol consumption, and exercise frequency was collected through questionnaires. Smoking status was classified as nonsmoker, former smoker, or current smoker. Mild alcohol consumption was defined as drinking < three times per week. Regular physical activity was classified as high-intensity physical activity (causing extreme shortness of breath) at least three times per week or moderate-intensity exercises (causing substantial shortness of breath) at least five times per week. Income levels were categorized into quartiles based on monthly earnings, with a focus on the lowest quartile proportion. BMI was calculated by dividing the weight of the participants in kilograms by their height in meters squared while glucose and lipid measurements were obtained after an overnight fast. Hypertension (HTN) was defined as meeting at least one of the following criteria per year: ICD-10 codes I10-13 or I15, in at least one claim per year for prescribed antihypertensive medication, or baseline systolic blood pressure (BP) ≥ 140 mmHg or diastolic BP ≥ 90 mmHg. Dyslipidemia was defined as meeting at least one of the following criteria per year: ICD-10 code E78 in claims, prescribed lipid-lowering medication, or baseline total cholesterol ≥ 240 mg/dL. CKD was defined as meeting the criteria at least once per year: ICD-10 code N18 or N19 in claims.

### Statistical analysis

The data are presented as the mean (standard deviation) for continuous variables and as numbers (percentages) for categorical variables. Kaplan–Meier curves were used to illustrate the cumulative incidence of incident DM and composite MACE, with group differences assessed using the log-rank test. Cox regression analyses were used to determine hazard ratios (HRs) and 95% confidence intervals (CIs) for outcome incidence rates. For multivariable-adjusted analyses, model 1 was adjusted for age and sex, while model 2 included additional adjustments for income, smoking status, alcohol consumption, regular physical activity, body weight (BW), HTN, dyslipidemia, and CKD. Relative excess risk due to interaction (RERI) and synergy index (SI), along with their 95% CIs were estimated [20]. A synergistic effect is considered evident when RERI and its 95% CI do not include 0, and when SI and its 95% CI do not include 1. A two-sided *P* < 0.05 indicated statistical significance in all analyses. Statistical analyses were performed using SAS version 9.4 (SAS Institute, Cary, NC, USA).

## Results

### Baseline characteristics by prediabetes status

The study included 896,585 young adults, of whom 57.4% were male, with a mean age of 30.6 years. Table 1 provides a summary of baseline characteristics based on prediabetes status. The median follow-up duration was 11.78 years (interquartile range [IQR], 1.65), similar across prediabetes status: 11.72 years (IQR, 1.67) for normoglycemia and 12.01 years (IQR, 1.27) for prediabetes. During follow-up 27,437 (3.1%) incident diabetes cases and 6,584 (0.7%) MACE cases were observed. There were 3,900 (0.4%) MI cases, 2,258 (0.3%) stroke cases, and 596 (0.1%) cardiovascular death cases.

**Table 1:** Baseline characteristics of study participants by prediabetes status

	Normoglycemia (*n* = 764,478)	Prediabetes (*n* = 132,107)
Age, years	30.3 ± 5.0	31.9 ± 4.8
Men	419,059 (54.8)	95,237 (72.1)
Income level, lowest 25%	167,649 (21.9)	27,983 (21.2)
Smoking		
Nonsmoker	462,609 (60.5)	63,701 (48.2)
Former smoker	69,837 (9.1)	15,838 (12.0)
Current smoker	232,032 (30.4)	52,568 (39.8)
Alcohol		
None	324,840 (42.5)	48,249 (36.5)
Mild	439,638 (57.5)	83,858 (63.5)
Regular physical activity	93,985 (12.3)	17,116 (13.0)
Body weight, kg	63.8 ± 13.0	69.5 ± 13.7
BMI, kg/m^2^	22.6 ± 3.4	24.0 ± 3.8
BMI		
< 18.5 kg/m^2^	66,045 (8.6)	5,793 (4.4)
18.5–22.9 kg/m^2^	387,982 (50.8)	49,569 (37.5)
23.0–24.9 kg/m^2^	142,593 (18.7)	27,993 (21.2)
25.0–29.9 kg/m^2^	144,622 (18.9)	39,798 (30.1)
≥ 30.0 kg/m^2^	23,236 (3.0)	8,954 (6.8)
Waist circumference		
In men	81.2 ± 8.1	83.4 ± 8.5
In women	70.1 ± 7.7	72.9 ± 9.3
SBP, mmHg	116.2 ± 12.6	121.6 ± 13.3
DBP, mmHg	72.8 ± 9.1	76.1 ± 9.6
Fasting glucose, mg/dL	86.2 ± 7.8	106.5 ± 6.1
Total cholesterol, mg/dL	182.0 ± 33.9	192.1 ± 38.4
Triglycerides, mg/dL	107.1 ± 81.6	139.8 ± 105.3
HDL-C, mg/dL	58.0 ± 28.0	55.4 ± 29.5
LDL-C, mg/dL	114.7 ± 269.5	122.9 ± 290.0
AST, IU/L	22.1 ± 16.5	24.7 ± 18.1
ALT, IU/L	22.0 ± 23.2	29.0 ± 28.5
GGT, IU/L	25.9 ± 25.3	36.8 ± 37.8
Hypertension	41,699 (5.5)	14,046 (10.6)
Dyslipidemia	55,627 (7.3)	16,141 (12.2)
Chronic kidney disease	205 (0.0)	48 (0.0)

Continuous variables are expressed as mean ± standard deviation. Categorical data are presented as frequencies and percentages. Abbreviations: BMI, body mass index; SBP, systolic blood pressure; DBP, diastolic blood pressure; AST, aspartate aminotransferase; ALT, alanine aminotransferase; GGT, gamma-glutamyl transferase.

### Prediabetes status and risk of cardiometabolic outcomes in young adults

**Fig. 1:**
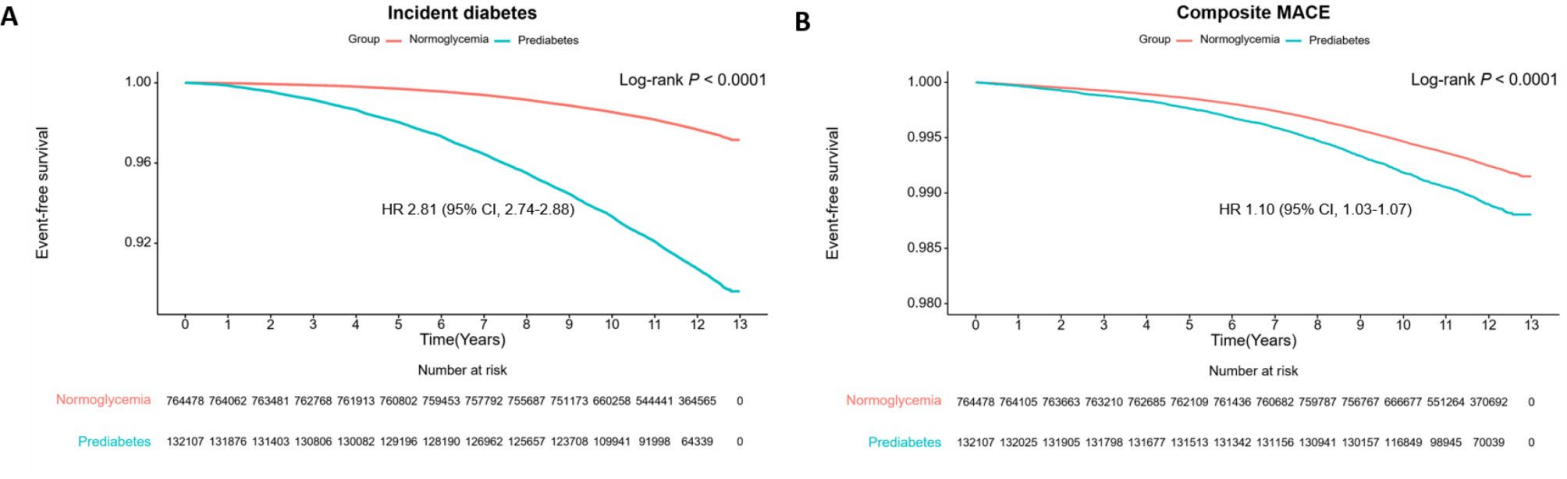
Kaplan–Meier estimates of cardiometabolic outcomes in young adults by prediabetes status. **(A)** Incident diabetes, **(B)** Major adverse cardiovascular events

Event-free survival for incident DM and composite MACE by prediabetes status, shown in Kaplan-Meier curves (Fig. 1**)**, indicated a significantly higher cumulative incidence of both outcomes among participants with prediabetes. As shown in Table 2, participants with prediabetes had a significantly increased risk of incident DM and composite MACE after adjusting for relevant covariates in model 2. Among the individual components of MACE, the cumulative incidence and risk of MI, stroke, and cardiovascular death was significantly higher among participants with prediabetes (Supplementary Fig. 2 and Supplementary Table 1). However, after adjusting for relevant covariates in model 2, the significance for stroke and cardiovascular death was lost.

**Table 2:** Incidence rates and hazard ratios for cardiometabolic outcomes in young adults by prediabetes status

	Event	Duration (person-years)	Incidence Rate^a^	Model 1		Model 2	
				Hazard Ratio (95% CI)	*P*-value	Hazard Ratio (95% CI)	*P*-value
Incident diabetes							
Normoglycemia	16,056	8,654,958	1.86	Reference		Reference	
Prediabetes	11,381	1,465,397	7.77	3.56 (3.47–3.65)	< 0.001	2.81 (2.74–2.88)	< 0.001
Composite MACE							
Normoglycemia	5,233	8,689,993	0.60	Reference		Reference	
Prediabetes	1,351	1,507,836	0.90	1.23 (1.15–1.30)	< 0.001	1.10 (1.03–1.17)	0.003

Abbreviations: CI, confidence interval; MACE, major adverse cardiovascular events. ^a^Incidence for 1000 person-years. Model 1: Adjusted for age and sex. Model 2: Adjusted for age, sex, income, smoking status, alcohol consumption, regular physical activity, body weight, hypertension, dyslipidemia, and chronic kidney disease.

### Effect of hepatic steatosis on cardiometabolic outcomes in young adults with prediabetes

**Fig. 2:**
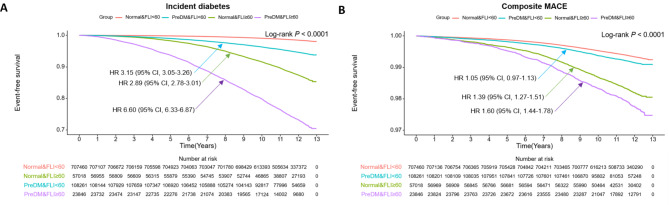
Kaplan–Meier estimates of cardiometabolic outcomes in young adults based on prediabetes and hepatic steatosis status. **(A)** Incident diabetes, **(B)** Major adverse cardiovascular events

Study participants were stratified based on hepatic steatosis using FLI (Supplementary Table 2). Among 764,478 individuals with normoglycemia and 132,107 with prediabetes, 57,018 (7.5%) and 23,846 (18.1%) had FLI-defined hepatic steatosis (FLI ≥ 60), respectively. Event-free survival for incident DM and composite MACE based on prediabetes and hepatic steatosis status, depicted in Kaplan-Meier curves (Fig. 2), showed the highest cumulative incidence of both outcomes in the prediabetes group with hepatic steatosis, followed by the normoglycemia group with hepatic steatosis, the prediabetes group without hepatic steatosis, and the normoglycemia without hepatic steatosis. Table 3 displays the HRs for incident diabetes and composite MACE based on prediabetes and hepatic steatosis status. After adjusting for relevant factors (model 2), the normoglycemia group with hepatic steatosis had a higher risk of incident DM and composite MACE than the normoglycemia group without hepatic steatosis. The coexistence of prediabetes and hepatic steatosis further increased the risk of incident DM and composite MACE, even after adjusting for relevant covariates (model 2). Additionally, the combination of prediabetes and hepatic steatosis additively increased the risk of DM and composite MACE (*P*_*trend*_ <0.001). A synergistic effect between prediabetes and hepatic steatosis on incident DM (RERI 9.93 [9.31–10.56], SI 1.92 [1.85–1.99], all *P*-value < 0.001) and composite MACE (RERI 0.44 [0.08–0.80], SI 1.22 [1.04–1.43], *P*-values 0.016 and 0.013, respectively) was also found. Among the individual components of MACE, the cumulative incidence and risk showed similar trends with those observed with composite MACE (Supplementary Fig. 3 and Supplementary Table 3).

**Table 3:** Incidence rates and hazard ratios for cardiometabolic outcomes in young adults based on prediabetes and h epatic steatosis status

	Event	Duration (person-years)	Incidence Rate^a^	Hazard Ratio (95% CI)			
				Model 1	*P*-value	Model 2	*P*-value
Incident diabetes							
Normoglycemia & FLI < 60	9,428	8,024,340	1.17	Reference		Reference	
Prediabetes & FLI < 60	5,383	1,218,812	4.42	3.52 (3.41–3.64)	< 0.001	3.15 (3.05–3.26)	< 0.001
Normoglycemia & FLI ≥ 60	6,628	630,618	10.51	8.06 (7.79–8.34)	< 0.001	2.89 (2.78–3.01)	< 0.001
Prediabetes & FLI ≥ 60	5,998	246,584	24.32	18.81 (18.16–19.48)	< 0.001	6.60 (6.33–6.87)	< 0.001
*P*_trend_					< 0.001		< 0.001
Composite MACE							
Normoglycemia & FLI < 60	4,282	8,039,555	0.53	Reference		Reference	
Prediabetes & FLI < 60	850	1,236,774	0.69	1.10 (1.02–1.19)	0.011	1.05 (0.97–1.13)	0.235
Normoglycemia & FLI ≥ 60	951	650,438	1.46	2.05 (1.91–2.20)	< 0.001	1.39 (1.27–1.51)	< 0.001
Prediabetes & FLI ≥ 60	501	271,063	1.85	2.46 (2.23–2.70)	< 0.001	1.60 (1.44–1.78)	< 0.001
*P*_trend_					< 0.001		< 0.001

Abbreviation: CI, confidence interval; MACE, major adverse cardiovascular events. ^a^Incidence for 1000 person-years. Model 1: Adjusted for age and sex. Model 2: Adjusted for age, sex, income, smoking status, alcohol consumption, regular physical activity, body weight, hypertension, dyslipidemia, and chronic kidney disease.

### Subgroup, sensitivity, and exploratory analyses

Subgroup analysis was conducted on incident DM and composite MACE by stratifying participants based on a baseline BMI value of 25 (Supplementary Table 4). The combined effect of prediabetes and hepatic steatosis on cardiometabolic outcomes was observed in both groups, but it was greater in the BMI < 25 kg/m^2^ group.

For the sensitivity analysis, we defined hepatic steatosis as FLI ≥ 30 (Supplementary Table 5). The combined effect of prediabetes and hepatic steatosis on cardiometabolic outcomes was similar to that in the main analysis (Supplementary Table 6). Additionally, we further analyzed the 5-year and 10-year cardiometabolic outcomes based on prediabetes and hepatic steatosis status (Supplementary Tables 7 and 8). The combined effect of prediabetes and hepatic steatosis on cardiometabolic outcomes was observed at both time points, but the effect diminished over time.

For the exploratory analysis, we investigated the effect of prediabetes and hepatic steatosis on liver-related outcomes including incident HCC and liver-related death (Supplementary Table 9). The prediabetes group with hepatic steatosis had a higher risk of incident HCC and liver-related death compared to the normoglycemia group without hepatic steatosis, after adjusting for relevant factors (model 2).

## Discussion

In this study, prediabetes was associated with an increased risk of incident DM and composite MACE in young adults, even after adjusting for relevant covariates. Moreover, the coexistence of prediabetes and hepatic steatosis was associated with an approximately 6.6-fold increase in the risk of incident DM and a 1.6-fold increase in the risk of composite MACE compared to those without either condition. Additionally, each condition additively contributed to the increased risk of cardiometabolic outcomes.

Prediabetes is recognized as a risk factor for developing CVD [2]. Previous studies have associated prediabetes with higher CVD risk in young adults. A study of Japanese young adults found that prediabetes increased the risk of MI, angina pectoris, and heart failure. The risk increased progressively with fasting glucose levels [6]. Additionally, a study in the Korean population found that transitioning from normoglycemia to prediabetes over two years was associated with an increased CVD risk [5]. In this study, the composite MACE risk was higher in the prediabetes group than in the normoglycemia group during the 11.8-year follow-up period in young adults, even after adjusting for relevant covariates, consistent with findings in previous studies. Hyperglycemia within a non-diabetic range seems to independently increase CVD risk in young adults. These findings are logical because prediabetes can cause increased oxidative stress, hyperinsulinemia, microvascular dysfunction, and chronic inflammation, all contributing to the pathophysiology of CVD [21, 22].

With the increasing prevalence of prediabetes among young adults [4] and the tendency for early-onset T2DM to lead to early cardiovascular complications [23], identifying individuals at high risk for T2DM and CVD within this demographic is crucial. Hepatic steatosis, identified as a high-risk subphenotype for T2DM and CVD among at-risk individuals [13], may serve as a predictive marker for cardiometabolic outcomes in young adults with prediabetes. Previous meta-analyses have revealed that individuals with hepatic steatosis are substantially more likely than those without the disease to develop T2DM and CVD [10–12]. Additionally, studies using FLI to assess hepatic steatosis have shown similar results, with an increased risk of cardiovascular events in the general population and T2DM patients with hepatic steatosis defined as FLI ≥ 60 [24–26]. Another study in the general population found a linear relationship between FLI and cardiovascular outcomes [27]. This study revealed that young adults with prediabetes and hepatic steatosis, defined by FLI ≥ 60, had a higher risk of cardiometabolic outcomes compared to individuals without these conditions. Furthermore, the combined presence of prediabetes and hepatic steatosis showed an additive effect on the risk of incident DM and composite MACE. These findings suggest that assessing hepatic steatosis could be a valuable strategy for identifying high-risk young adults with prediabetes for cardiometabolic outcomes.

Insulin resistance (IR) and chronic inflammation are proposed as potential mechanisms linking hepatic steatosis with CVD [11, 28]. Increased free fatty acid levels in circulation correlate with IR, resulting in ectopic fat buildup in the liver and epicardium [29]. Epicardial fat accumulation is associated with hepatic steatosis [30, 31] and adverse cardiometabolic outcomes [32]. Therefore, IR is pivotal in linking cardiometabolic disease (CMD) and steatotic liver disease (SLD). Chronic inflammation, alongside IR, also contributes to connecting SLD and cardiometabolic disease (CMD). Systemic inflammation usually accompanies hepatic inflammation as SLD advances [33, 34]. Chronic inflammation potentially links SLD and CMD by affecting all atherosclerosis stages, leading to CMD [35, 36]. However, the influence of IR and systemic inflammation on the hepatic steatosis-CMD link could not be determined in this study, as these factors were not assessed in the study participants. Therefore, further research is warranted to investigate whether IR and chronic inflammation mediate the hepatic steatosis-CMD relationship in young adults with prediabetes.

Both prediabetes and hepatic steatosis are associated with an increased risk of intra- and extra-hepatic cancers [37–39]. It is well established that prediabetes and hepatic steatosis independently contribute to increased oxidative stress [40, 41], which plays a critical role in cancer evolution [42]. Therefore, when prediabetes and hepatic steatosis coexist, the cancer risk may increase in an additive manner. In our exploratory analysis, we observed that the risk of incident HCC was significantly higher in individuals with prediabetes with hepatic steatosis compared to those with normoglycemia without hepatic steatosis. However, we were unable to assess whether this increased risk was attributable to elevated oxidative stress, as oxidative stress biomarkers were not available. Moreover, we did not evaluate whether the coexistence of prediabetes and hepatic steatosis also increases the risk of extra-hepatic cancers. Further research is needed to investigate whether the combination of prediabetes and hepatic steatosis leads to an enhanced risk of intra- and extra-hepatic cancers through mechanisms involving oxidative stress.

This study had several limitations. First, only fasting glucose was used because the NHIS claims database lacked information about glycated hemoglobin (HbA1c) and 75 g oral glucose tolerance test (OGTT). Although several guidelines suggest including HbA1c and 2-h postprandial glucose during a 75 g OGTT for diagnosing prediabetes [2, 43], this data was not available. Second, since the majority of participants (up to 75%) did not have follow-up fasting glucose data within one year, we were unable to investigate the association between changes in fasting glucose and cardiometabolic outcomes. Third, hepatic steatosis was assessed solely with the FLI, without radiologic confirmation or biopsy. While FLI is used to assess hepatic steatosis in various large-population studies based on claims data, it cannot differentiate between steatosis, steatohepatitis, and fibrosis [24–26, 44]. In addition, FLI may have differences in diagnostic accuracy across weight strata [45] and may also have lower cutoff values among Asians [46]. However, in our subgroup and sensitivity analyses, the combined effect of prediabetes and hepatic steatosis on cardiometabolic outcomes was similar to that observed in the main analysis. Fourth, we were unable to further stratify our study participants according to steatohepatitis or fibrosis risk using the fibrotic non-alcoholic steatohepatitis index [47] or the metabolic dysfunction-associated fibrosis 5 score [48], both of which are developed for the general population, because HbA1c and platelet count data were not available in our dataset. Fifth, since the primary aim of this study was to investigate the effect of hepatic steatosis on young adults with prediabetes, given that this population is known to experience earlier progression of complications when diagnosed with diabetes [49], we only included adults younger than 40 years. Therefore, our results cannot be generalized to middle-aged or older adults. Sixth, since due to the absence of smoking duration data in the dataset, only smoking status—categorized as nonsmoker, former smoker, and current smoker—was adjusted as a covariate. Seventh, despite initially excluding patients with prior diabetes and cardiovascular events to minimize reverse causality, the study could not establish causation. Finally, as the study population was limited to a single country (Korea), the results may not be applicable to other ethnic groups.

In conclusion, prediabetes and hepatic steatosis additively increased the risk of cardiometabolic outcomes in young adults. These findings emphasize the relevance of including hepatic steatosis screening in the CMD risk assessment for young adults with prediabetes. 

## Electronic supplementary material

Below is the link to the electronic supplementary material.

Supplementary Material 1

## Data Availability

No datasets were generated or analysed during the current study.
