# Supplementary material for: Combined impact of prediabetes and hepatic steatosis on cardiometabolic outcomes in young adults
Source: Cardiovasc Diabetol. 2024 Nov 21;23:422. doi: 10.1186/s12933-024-02516-4 (PMC11583572; doi:10.1186/s12933-024-02516-4)
Supplement: Supplementary file 1 — Supplementary Material 1 [file 12933_2024_2516_MOESM1_ESM.docx]

**Supplementary Figure 1.** Study flow chart


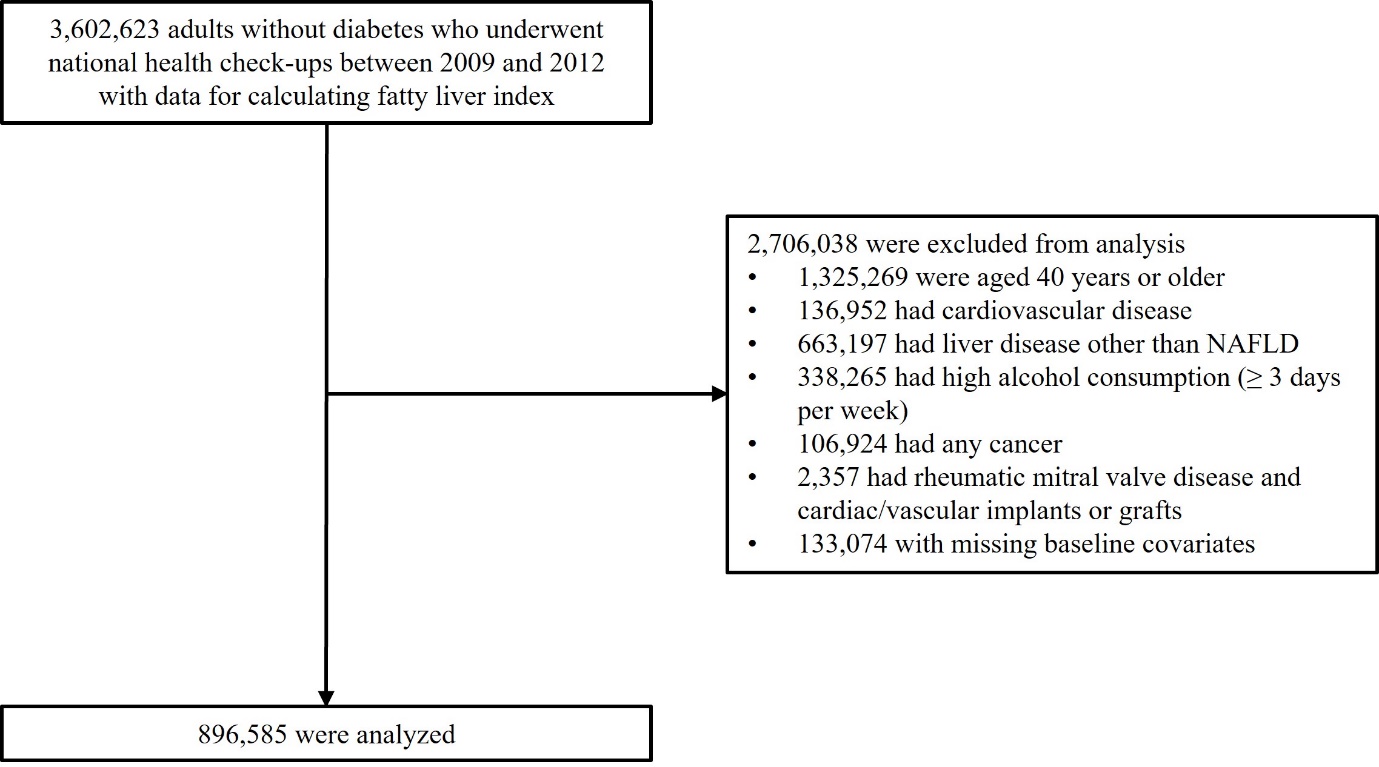


**Supplementary Figure 2.** Kaplan–Meier estimates of individual cardiovascular outcomes in young adults by prediabetes status. **A.** Myocardial infarction, **B.** Stroke, and **C**. Cardiovascular death


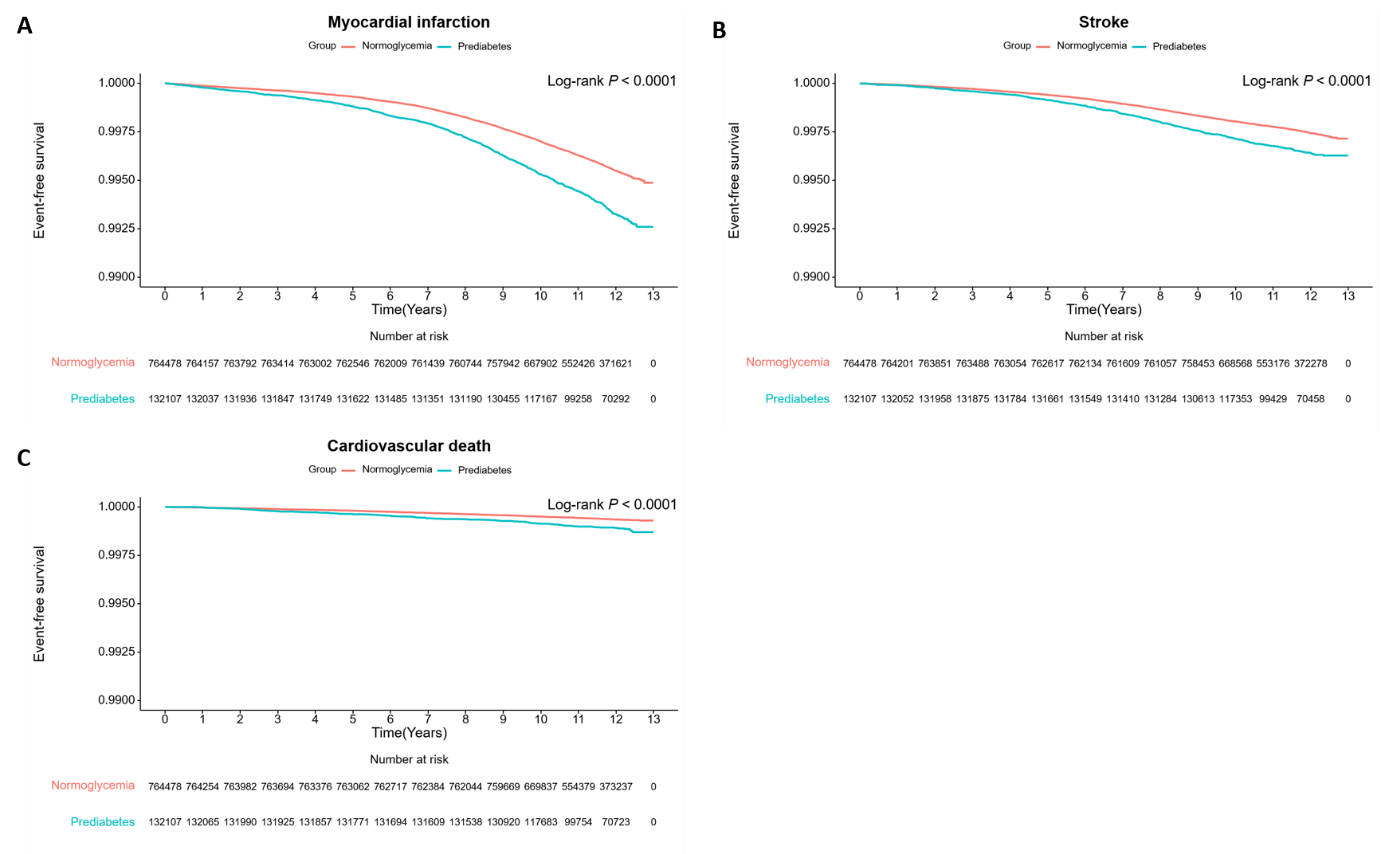


**Supplementary Figure 3.** Kaplan–Meier estimates of individual cardiovascular outcomes in young adults by prediabetes and hepatic steatosis status. **A.** Myocardial infarction, **B.** Stroke, and **C**. Cardiovascular death


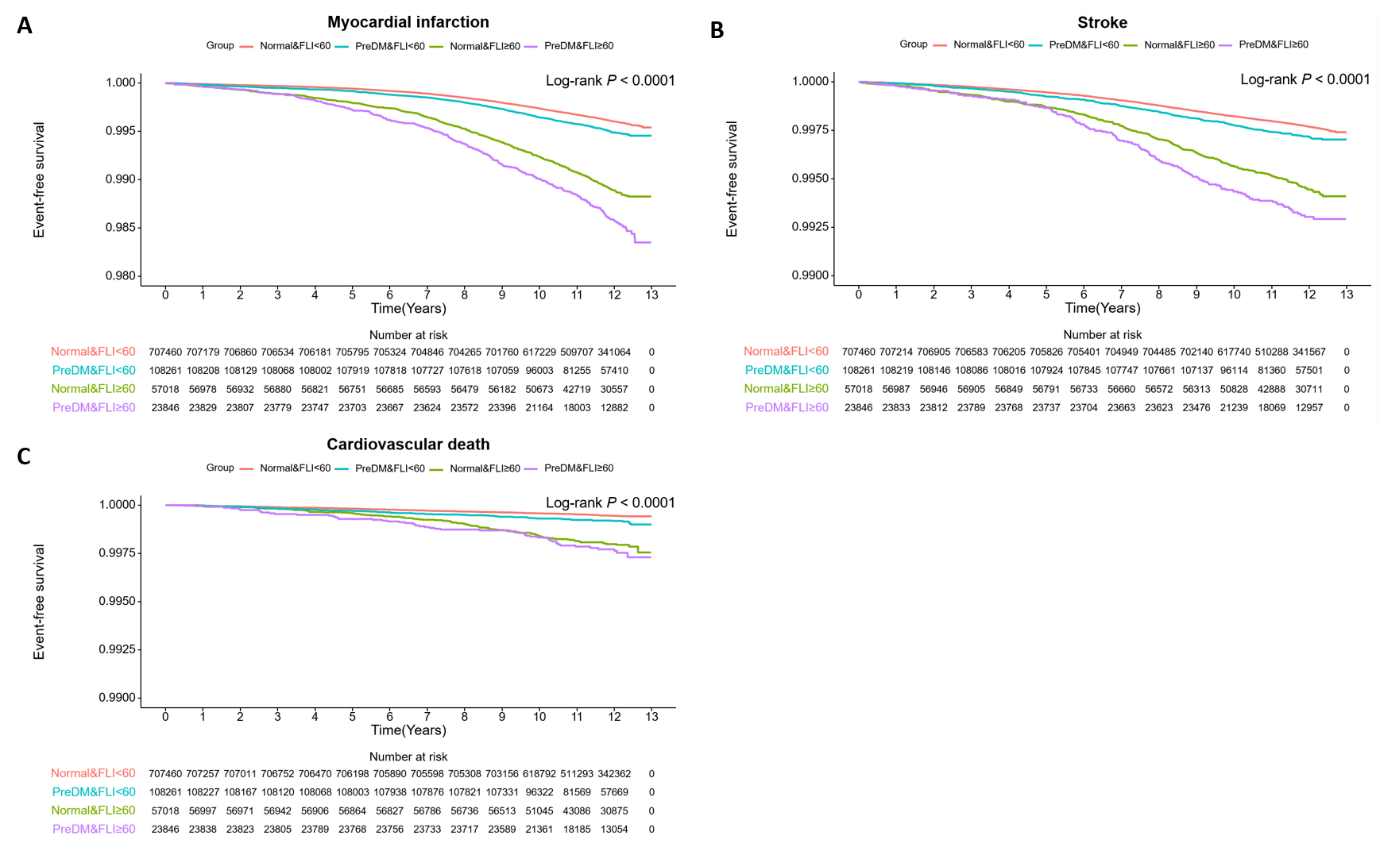


| **Supplementary Table 1.** Incidence rates and hazard ratios for individual cardiovascular outcomes in young adults by prediabetes status | | | | | | | | |  |
| --- | --- | --- | --- | --- | --- | --- | --- | --- | --- |
|  |  | Event | Duration (person-years) | Incidence Rate^a^ | Model 1 | | Model 2 | | |
|  |  |  |  |  | Hazard Ratio (95% CI) | *P*-value | Hazard Ratio (95% CI) | *P*-value | |
| Myocardial infarction |  |  |  |  |  |  |  |  | |
| Normoglycemia |  | 3,089 | 8,697,602 | 0.36 | Reference |  | Reference |  | |
| Prediabetes |  | 811 | 1,509,794 | 0.54 | 1.26 (1.17-1.36) | <0.001 | 1.14 (1.05-1.23) | 0.002 | |
| Stroke |  |  |  |  |  |  |  |  | |
| Normoglycemia |  | 1,813 | 8,700,870 | 0.21 | Reference |  | Reference |  | |
| Prediabetes |  | 445 | 1,510,750 | 0.29 | 1.16 (1.05-1.29) | 0.005 | 1.04 (0.94-1.16) | 0.428 | |
| Cardiovascular death |  |  |  |  |  |  |  |  | |
| Normoglycemia |  | 458 | 8,708,704 | 0.05 | Reference |  | Reference |  | |
| Prediabetes |  | 138 | 1,512,765 | 0.09 | 1.30 (1.07-1.57) | 0.008 | 1.10 (0.90-1.33) | 0.355 | |

Abbreviations: CI, confidence interval; MACE, major adverse cardiovascular events. ^a^Incidence for 1000 person-years. Model 1: Adjusted for age and sex. Model 2: Adjusted for age, sex, income, smoking status, alcohol consumption, regular physical activity, body weight, hypertension, dyslipidemia, and chronic kidney disease.

| **Supplementary Table 2.** Baseline characteristics of study participants based on prediabetes and hepatic steatosis status | | | | | | |
| --- | --- | --- | --- | --- | --- | --- |
|  |  | Normoglycemia & FLI < 60 (*n*=707,460) | Prediabetes & FLI < 60 (*n*=108,261) | Normoglycemia & FLI ≥ 60 (*n*=57,018) | Prediabetes & FLI ≥ 60 (*n*=23,846) | *P*-value |
| Age, years |  | 30.2 ± 5.0 | 31.6 ± 4.9 | 32.3 ± 4.3 | 31.6 ± 4.9 | <0.001 |
| Male |  | 365,687 (51.7) | 72,940 (67.4) | 53,372 (93.6) | 22,297 (93.5) | <0.001 |
| Income level, lowest 25% |  | 158,887 (22.5) | 24,252 (22.4) | 8,762 (15.4) | 3,731 (15.7) | <0.001 |
| Smoking |  |  |  |  |  | <0.001 |
| Nonsmoker |  | 446,646 (63.1) | 57,147 (52.8) | 15,963 (28.0) | 6,554 (27.5) |  |
| Former smoker |  | 62,289 (8.8) | 12,412 (11.5) | 7,548 (13.2) | 3,426 (14.4) |  |
| Current smoker |  | 198,525 (28.1) | 38,702 (35.8) | 33,507 (58.8) | 13,866 (58.2) |  |
| Alcohol |  |  |  |  |  | <0.001 |
| None |  | 309,528 (43.8) | 42,256 (39.0) | 15,312 (26.9) | 5,993 (25.1) |  |
| Mild |  | 397,932 (56.3) | 66,005 (61.0) | 41,706 (73.2) | 17,853 (74.9) |  |
| Regular physical activity |  | 86,594 (12.2) | 14,191 (13.1) | 7,391 (13.0) | 2,925 (12.3) | <0.001 |
| Body weight, kg |  | 62.0 ± 11.3 | 65.8 ± 11.1 | 86.4 ± 11.1 | 86.4 ± 11.4 | <0.001 |
| BMI |  |  |  |  |  | <0.001 |
| < 18.5 kg/m^2^ |  | 66,038 (9.3) | 5,793 (5.4) | 7 (0.0) | 0 (0.0) |  |
| 18.5-22.9 kg/m^2^ |  | 387,357 (54.8) | 49,331 (45.6) | 625 (1.1) | 238 (1.0) |  |
| 23.0-24.9 kg/m^2^ |  | 138,591 (19.6) | 26,275 (24.3) | 4,002 (7.0) | 1,718 (7.2) |  |
| 25.0-29.9 kg/m^2^ |  | 110,184 (15.6) | 25,545 (23.6) | 34,438 (60.4) | 14,253 (59.8) |  |
| ≥ 30.0 kg/m^2^ |  | 5,290 (0.8) | 1,317 (1.2) | 17,946 (31.5) | 7,637 (32.0) |  |
| Waist circumference |  |  |  |  |  |  |
| In men |  | 79.5 ± 6.7 | 80.5 ± 6.5 | 92.8 ± 7.3 | 92.9 ± 7.4 | <0.001 |
| In women |  | 69.8 ± 7.3 | 71.9 ± 7.9 | 95.7 ± 8.8 | 95.6 ± 9.1 | <0.001 |
| SBP, mmHg |  | 115.3 ± 12.2 | 119.9 ± 12.6 | 127.4 ± 12.9 | 129.5 ± 13.6 | <0.001 |
| DBP, mmHg |  | 72.2 ± 8.7 | 74.9 ± 9.0 | 80.4 ± 9.5 | 81.8 ± 10.1 | <0.001 |
| Fasting glucose, mg/dL |  | 86.0 ± 7.8 | 106.2 ± 5.9 | 88.1 ± 7.6 | 107.8 ± 6.5 | <0.001 |
| Total cholesterol, mg/dL |  | 179.7 ± 32.7 | 187.3 ± 36.8 | 209.6 ± 36.3 | 214.1 ± 38.1 | <0.001 |
| Triglycerides, mg/dL |  | 95.8 ± 59.4 | 113.5 ± 66.3 | 247.5 ± 155.7 | 259.3 ± 155.0 | <0.001 |
| HDL-C, mg/dL |  | 58.7 ± 27.1 | 56.9 ± 29.7 | 49.3 ± 37.0 | 48.6 ± 27.5 | <0.001 |
| LDL-C, mg/dL |  | 114.1 ± 275.6 | 123.1 ± 309.6 | 122.5 ± 178.7 | 121.9 ± 175.3 | <0.001 |
| AST, IU/L |  | 21.3 ± 15.6 | 22.5 ± 12.5 | 32.6 ± 22.6 | 34.7 ± 31.3 | <0.001 |
| ALT, IU/L |  | 19.7 ± 18.8 | 23.4 ± 20.5 | 50.6 ± 44.3 | 54.5 ± 42.4 | <0.001 |
| GGT, IU/L |  | 22.2 ± 16.7 | 27.4 ± 21.1 | 71.0 ± 53.9 | 79.6 ± 60.4 | <0.001 |
| Hypertension |  | 31,021 (4.38) | 8,403 (7.8) | 10,678 (18.7) | 5,643 (23.7) | <0.001 |
| Dyslipidemia |  | 42,087 (5.6) | 9,620 (8.9) | 13,540 (23.8) | 6,521 (27.4) | <0.001 |
| Chronic kidney disease |  | 181 (0.0) | 41 (0.0) | 24 (0.0) | 7 (0.0) | <0.001 |

Continuous variables are expressed as mean ± standard deviation. Categorical data are presented as frequencies and percentages. Abbreviations: BMI, body mass index; SBP, systolic blood pressure; DBP, diastolic blood pressure; HDL-C, high-density lipoprotein cholesterol; LDL-C, low-density lipoprotein cholesterol; AST, aspartate aminotransferase; ALT, alanine aminotransferase; GGT, gamma-glutamyl transferase.

| **Supplementary Table 3.** Incidence rates and hazard ratios for individual cardiovascular outcomes in young adults based on prediabetes and hepatic steatosis status | | | | | | | | |  |
| --- | --- | --- | --- | --- | --- | --- | --- | --- | --- |
|  |  | Event | Duration (person-years) | Incidence Rate^a^ | Hazard Ratio (95% CI) | | | | |
|  |  |  |  |  | Model 1 | *P*-value | Model 2 | *P*-value | |
| Myocardial infarction |  |  |  |  |  |  |  |  | |
| Normoglycemia & FLI < 60 |  | 2,513 | 8,045,910 | 0.31 | Reference |  | Reference |  | |
| Prediabetes & FLI < 60 |  | 498 | 1,238,045 | 0.40 | 1.11 (1.01-1.22) | 0.032 | 1.07 (0.97-1.17) | 0.200 | |
| Normoglycemia & FLI ≥ 60 |  | 576 | 651,692 | 0.88 | 2.13 (1.94-2.34) | <0.001 | 1.49 (1.34-1.67) | <0.001 | |
| Prediabetes & FLI ≥ 60 |  | 313 | 271,749 | 1.15 | 2.67 (2.36-3.01) | <0.001 | 1.82 (1.59-2.08) | <0.001 | |
| *P*_trend_ |  |  |  |  |  | <0.001 |  | <0.001 | |
| Stroke |  |  |  |  |  |  |  |  | |
| Normoglycemia & FLI < 60 |  | 1,516 | 8,048,292 | 0.19 | Reference |  | Reference |  | |
| Prediabetes & FLI < 60 |  | 290 | 1,238,538 | 0.23 | 1.06 (0.93-1.20) | 0.389 | 1.00 (0.88-1.13) | 0.994 | |
| Normoglycemia & FLI ≥ 60 |  | 297 | 652,577 | 0.46 | 1.84 (1.62-2.09) | <0.001 | 1.26 (1.08-1.47) | 0.003 | |
| Prediabetes & FLI ≥ 60 |  | 155 | 272,212 | 0.57 | 2.15 (1.81-2.54) | <0.001 | 1.40 (1.16-1.69) | 0.001 | |
| *P*_trend_ |  |  |  |  |  | <0.001 |  | <0.001 | |
| Cardiovascular death |  |  |  |  |  |  |  |  | |
| Normoglycemia & FLI < 60 |  | 348 | 8,054,814 | 0.04 | Reference |  | Reference |  | |
| Prediabetes & FLI < 60 |  | 85 | 1,239,845 | 0.07 | 1.24 (0.97-1.57) | 0.083 | 1.13 (0.89-1.43) | 0.320 | |
| Normoglycemia & FLI ≥ 60 |  | 110 | 653,890 | 0.17 | 2.42 (1.94-3.02) | <0.001 | 1.28 (0.98-1.68) | 0.068 | |
| Prediabetes & FLI ≥ 60 |  | 53 | 272,920 | 0.19 | 2.60 (1.93-3.49) | <0.001 | 1.29 (0.92-1.80) | 0.138 | |
| *P*_trend_ |  |  |  |  |  | <0.001 |  | 0.049 | |

Abbreviation: CI, confidence interval; MACE, major adverse cardiovascular events. ^a^Incidence for 1000 person-years. Model 1: Adjusted for age and sex. Model 2: Adjusted for age, sex, income, smoking status, alcohol consumption, regular physical activity, body weight, hypertension, dyslipidemia, and chronic kidney disease.

| **Supplementary Table 4.** Incidence rates and hazard ratios for cardiometabolic outcomes in young adults based on prediabetes and hepatic steatosis status (BMI < 25 vs. ≥ 25 kg/m^2^) | | | | | | |  |
| --- | --- | --- | --- | --- | --- | --- | --- |
|  |  | Event | Duration (person-years) | Incidence Ratea | Hazard Ratio (95% CI)^b^ | *P* for interaction | |
| Incident diabetes |  |  |  |  |  | <0.001 | |
| BMI < 25 kg/m2 |  |  |  |  |  |  | |
| Normoglycemia & FLI < 60 |  | 5,122 | 6,722,562 | 0.76 | Reference |  | |
| Prediabetes & FLI < 60 |  | 2,656 | 922,472 | 2.88 | 3.13 (2.99-3.29) |  | |
| Normoglycemia & FLI ≥ 60 |  | 313 | 52,798 | 5.93 | 3.32 (2.95-3.74) |  | |
| Prediabetes & FLI ≥ 60 |  | 342 | 21,219 | 16.12 | 8.85 (7.88-9.93) |  | |
| BMI ≥ 25 kg/m2 |  |  |  |  |  |  | |
| Normoglycemia & FLI < 60 |  | 4,306 | 1,301,778 | 3.31 | Reference |  | |
| Prediabetes & FLI < 60 |  | 2,727 | 296,340 | 9.20 | 2.75 (2.62-2.88) |  | |
| Normoglycemia & FLI ≥ 60 |  | 6,315 | 577,820 | 10.93 | 2.48 (2.38-2.59) |  | |
| Prediabetes & FLI ≥ 60 |  | 5,656 | 225,366 | 25.10 | 5.73 (5.48-5.99) |  | |
| Composite MACE |  |  |  |  |  | 0.003 | |
| BMI < 25 kg/m2 |  |  |  |  |  |  | |
| Normoglycemia & FLI < 60 |  | 3,254 | 6,728,058 | 0.48 | Reference |  | |
| Prediabetes & FLI < 60 |  | 566 | 930,829 | 0.61 | 1.02 (0.93-1.12) |  | |
| Normoglycemia & FLI ≥ 60 |  | 62 | 53,594 | 1.16 | 1.24 (0.96-1.61) |  | |
| Prediabetes & FLI ≥ 60 |  | 45 | 22,458 | 2.00 | 1.97 (1.46-2.66) |  | |
| BMI ≥ 25 kg/m2 |  |  |  |  |  |  | |
| Normoglycemia & FLI < 60 |  | 1,028 | 1,311,497 | 0.78 | Reference |  | |
| Prediabetes & FLI < 60 |  | 284 | 305,944 | 0.93 | 1.07 (0.94-1.22) |  | |
| Normoglycemia & FLI ≥ 60 |  | 889 | 596,844 | 1.49 | 1.38 (1.25-1.53) |  | |
| Prediabetes & FLI ≥ 60 |  | 456 | 248,605 | 1.83 | 1.57 (1.39-1.78) |  | |
| Myocardial infarction |  |  |  |  |  | 0.362 | |
| BMI < 25 kg/m2 |  |  |  |  |  |  | |
| Normoglycemia & FLI < 60 |  | 1,920 | 6,732,924 | 0.29 | Reference |  | |
| Prediabetes & FLI < 60 |  | 335 | 931,686 | 0.36 | 1.05 (0.93-1.18) |  | |
| Normoglycemia & FLI ≥ 60 |  | 40 | 53,663 | 0.75 | 1.42 (1.03-1.95) |  | |
| Prediabetes & FLI ≥ 60 |  | 21 | 22,541 | 0.93 | 1.67 (1.08-2.59) |  | |
| BMI ≥ 25 kg/m2 |  |  |  |  |  |  | |
| Normoglycemia & FLI < 60 |  | 593 | 1,312,986 | 0.45 | Reference |  | |
| Prediabetes & FLI < 60 |  | 163 | 306,359 | 0.53 | 1.08 (0.90-1.28) |  | |
| Normoglycemia & FLI ≥ 60 |  | 536 | 598,030 | 0.90 | 1.49 (1.30-1.70) |  | |
| Prediabetes & FLI ≥ 60 |  | 292 | 249,208 | 1.17 | 1.82 (1.55-2.13) |  | |
| Stroke |  |  |  |  |  | 0.002 | |
| BMI < 25 kg/m2 |  |  |  |  |  |  | |
| Normoglycemia & FLI < 60 |  | 1,154 | 6,734,719 | 0.17 | Reference |  | |
| Prediabetes & FLI < 60 |  | 194 | 932,023 | 0.21 | 0.97 (0.83-1.13) |  | |
| Normoglycemia & FLI ≥ 60 |  | 18 | 53,742 | 0.33 | 1.01 (0.63-1.62) |  | |
| Prediabetes & FLI ≥ 60 |  | 20 | 22,547 | 0.89 | 2.34 (1.49-3.68) |  | |
| BMI ≥ 25 kg/m2 |  |  |  |  |  |  | |
| Normoglycemia & FLI < 60 |  | 362 | 1,313,574 | 0.28 | Reference |  | |
| Prediabetes & FLI < 60 |  | 96 | 306,515 | 0.31 | 1.02 (0.81-1.28) |  | |
| Normoglycemia & FLI ≥ 60 |  | 279 | 598,835 | 0.47 | 1.31 (1.09-1.57) |  | |
| Prediabetes & FLI ≥ 60 |  | 135 | 249,665 | 0.54 | 1.37 (1.10-1.71) |  | |
| Cardiovascular death |  |  |  |  |  | 0.143 | |
| BMI < 25 kg/m2 |  |  |  |  |  |  | |
| Normoglycemia & FLI < 60 |  | 245 | 6,739,710 | 0.04 | Reference |  | |
| Prediabetes & FLI < 60 |  | 52 | 932,893 | 0.06 | 1.08 (0.80-1.47) |  | |
| Normoglycemia & FLI ≥ 60 |  | 9 | 53,827 | 0.17 | 1.82 (0.92-3.62) |  | |
| Prediabetes & FLI ≥ 60 |  | 4 | 22,629 | 0.18 | 1.64 (0.60-4.47) |  | |
| BMI ≥ 25 kg/m2 |  |  |  |  |  |  | |
| Normoglycemia & FLI < 60 |  | 103 | 1,315,105 | 0.08 | Reference |  | |
| Prediabetes & FLI < 60 |  | 33 | 306,951 | 0.11 | 1.19 (0.80-1.76) |  | |
| Normoglycemia & FLI ≥ 60 |  | 101 | 600,063 | 0.17 | 1.11 (0.81-1.52) |  | |
| Prediabetes & FLI ≥ 60 |  | 49 | 250,290 | 0.20 | 1.15 (0.78-1.67) |  | |

Abbreviation: CI, confidence interval; MACE, major adverse cardiovascular events; BMI, body mass index. ^a^Incidence for 1000 person-years. ^b^Adjusted for age, sex, income, smoking status, alcohol consumption, regular physical activity, body weight, hypertension, dyslipidemia, and chronic kidney disease.

smoking status, alcohol consumption, regular physical activity, body weight, hypertension, dyslipidemia, and chronic kidney disease.

| **Supplementary Table 5.** Baseline characteristics of study participants based on prediabetes and hepatic steatosis status | | | | | | |
| --- | --- | --- | --- | --- | --- | --- |
|  |  | Normoglycemia & FLI < 30  (*n*=600,999) | Prediabetes  & FLI < 30  (*n*=78,962) | Normoglycemia & FLI ≥ 30  (*n*=163,479) | Prediabetes & FLI ≥ 30  (*n*=53,145) | *P*-value |
| Age, years |  | 29.8 ± 5.0 | 31.1 ± 5.0 | 32.1 ± 4.4 | 33.1 ± 4.2 | <0.001 |
| Male |  | 270,305 (45.0) | 46,601 (59.0) | 148,754 (90.1) | 48,636 (91.5) | <0.001 |
| Income level, lowest 25% |  | 141,408 (23.5) | 19,281 (24.4) | 26,241 (16.1) | 8,702 (16.4) | <0.001 |
| Smoking |  |  |  |  |  | <0.001 |
| Nonsmoker |  | 407,945 (67.9) | 46,783 (59.3) | 54,664 (33.4) | 16,918 (31.8) |  |
| Former smoker |  | 47,208 (7.9) | 7,770 (9.8) | 22,629 (13.8) | 8,068 (15.2) |  |
| Current smoker |  | 145,846 (24.3) | 24,409 (30.9) | 86,186 (52.7) | 28,159 (53.0) |  |
| Alcohol |  |  |  |  |  | <0.001 |
| None |  | 276,412 (46.0) | 33,598 (42.6) | 48,428 (29.6) | 14,651 (27.6) |  |
| Mild |  | 324,587 (54.0) | 45,364 (57.5) | 115,051 (70.4) | 38,494 (72.4) |  |
| Regular physical activity |  | 72,187 (12.0) | 10,128 (12.8) | 21,798 (13.3) | 6,988 (13.2) | <0.001 |
| Body weight, kg |  | 59.5 ± 9.8 | 62.1 ± 9.7 | 79.7 ± 10.6 | 80.5 ± 11.1 | <0.001 |
| BMI |  |  |  |  |  | <0.001 |
| < 18.5 kg/m^2^ |  | 65,994 (11.0) | 5,776 (7.3) | 51 (0.0) | 17 (0.1) |  |
| 18.5-22.9 kg/m^2^ |  | 377,477 (62.8) | 46,466 (58.9) | 10,505 (6.4) | 3,103 (5.8) |  |
| 23.0-24.9 kg/m^2^ |  | 107,785 (17.9) | 17,782 (22.5) | 34,808 (21.3) | 10,211 (19.2) |  |
| 25.0-29.9 kg/m^2^ |  | 48,961 (8.2) | 8,798 (11.1) | 95,661 (58.5) | 31,000 (58.3) |  |
| ≥ 30.0 kg/m^2^ |  | 782 (0.1) | 140 (0.2) | 22,454 (13.7) | 8,814 (16.6) |  |
| Waist circumference |  |  |  |  |  |  |
| In men |  | 77.3 ± 5.8 | 77.8 ± 5.6 | 88.2 ± 6.9 | 88.8 ± 7.3 | <0.001 |
| In women |  | 69.3 ± 6.6 | 70.7 ± 6.8 | 88.5 ± 8.5 | 88.8 ± 9.0 | <0.001 |
| SBP, mmHg |  | 114.1 ± 11.8 | 118.1 ± 12.3 | 124.2 ± 12.4 | 126.9 ± 13.1 | <0.001 |
| DBP, mmHg |  | 71.3 ± 8.5 | 73.7 ± 8.8 | 78.1 ± 9.1 | 79.8 ± 9.6 | <0.001 |
| Fasting glucose, mg/dL |  | 85.7 ± 7.7 | 106.0 ± 5.8 | 87.7 ± 7.7 | 107.3 ± 6.3 | <0.001 |
| Total cholesterol, mg/dL |  | 176.6 ± 31.3 | 182.0 ± 33.9 | 201.7 ± 35.7 | 207.0 ± 39.8 | <0.001 |
| Triglycerides, mg/dL |  | 83.4 ± 43.1 | 93.3 ± 45.7 | 194.2 ± 121.0 | 208.9 ± 128.4 | <0.001 |
| HDL-C, mg/dL |  | 59.9 ± 25.2 | 58.8 ± 22.0 | 50.9 ± 35.7 | 50.3 ± 37.5 | <0.001 |
| LDL-C, mg/dL |  | 112.9 ± 291.9 | 122.4 ± 342.1 | 121.5 ± 162.5 | 123.6 ± 187.5 | <0.001 |
| AST, IU/L |  | 20.4 ± 12.8 | 21.1 ± 11.1 | 28.3 ± 24.9 | 30.1 ± 24.1 | <0.001 |
| ALT, IU/L |  | 17.4 ± 14.9 | 19.4 ± 15.6 | 39.0 ± 36.5 | 43.3 ± 36.3 | <0.001 |
| GGT, IU/L |  | 18.9 ± 11.0 | 21.3 ± 12.8 | 51.5 ± 41.2 | 59.7 ± 49.2 | <0.001 |
| Hypertension |  | 20,852 (3.5) | 4,620 (5.9) | 20,847 (12.8) | 9,426 (17.7) | <0.001 |
| Dyslipidemia |  | 27,587 (4.6) | 4,888 (6.2) | 28,040 (17.2) | 11,253 (21.2) | <0.001 |
| Chronic kidney disease |  | 144 (0.2) | 27 (0.0) | 61 (0.0) | 21 (0.0) | 0.007 |

Continuous variables are expressed as mean ± standard deviation. Categorical data are presented as frequencies and percentages. Abbreviations: BMI, body mass index; SBP, systolic blood pressure; DBP, diastolic blood pressure; HDL-C, high-density lipoprotein cholesterol; LDL-C, low-density lipoprotein cholesterol; AST, aspartate aminotransferase; ALT, alanine aminotransferase; GGT, gamma-glutamyl transferase.

| **Supplementary Table 6.** Incidence rates and hazard ratios for cardiometabolic outcomes in young adults based on prediabetes and hepatic steatosis status (FLI < 30 vs. FLI ≥ 30) | | | | | | | | |  |
| --- | --- | --- | --- | --- | --- | --- | --- | --- | --- |
|  |  | Event | Duration (person-years) | Incidence Rate^a^ | Hazard Ratio (95% CI) | | | | |
|  |  |  |  |  | Model 1 | *P*-value | Model 2 | *P*-value | |
| Incident diabetes |  |  |  |  |  |  |  |  | |
| Normoglycemia & FLI < 30 |  | 5,123 | 6,815,589 | 0.75 | Reference |  | Reference |  | |
| Prediabetes & FLI < 30 |  | 2,282 | 894,757 | 2.55 | 3.42 (3.25-3.59) | <0.001 | 3.12 (2.97-3.28) | <0.001 | |
| Normoglycemia & FLI ≥ 30 |  | 10,933 | 1,839,369 | 5.94 | 8.63 (8.31-8.96) | <0.001 | 3.59 (3.44-3.74) | <0.001 | |
| Prediabetes & FLI ≥ 30 |  | 9,099 | 570,640 | 15.95 | 23.31 (22.43-24.23) | <0.001 | 9.05 (8.67-9.45) | <0.001 | |
| *P*_trend_ |  |  |  |  |  | <0.001 |  | <0.001 | |
| Composite MACE |  |  |  |  |  |  |  |  | |
| Normoglycemia & FLI < 30 |  | 3,188 | 6,821,243 | 0.47 | Reference |  | Reference |  | |
| Prediabetes & FLI < 30 |  | 512 | 901,577 | 0.57 | 1.06 (0.97-1.17) | 0.189 | 1.03 (0.93-1.13) | 0.601 | |
| Normoglycemia & FLI ≥ 30 |  | 2,045 | 1,868,749 | 1.09 | 1.75 (1.64-1.85) | <0.001 | 1.28 (1.19-1.37) | <0.001 | |
| Prediabetes & FLI ≥ 30 |  | 839 | 606,259 | 1.38 | 2.09 (1.93-2.27) | <0.001 | 1.44 (1.31-1.58) | <0.001 | |
| *P*_trend_ |  |  |  |  |  | <0.001 |  | <0.001 | |
| Myocardial infarction |  |  |  |  |  |  |  |  | |
| Normoglycemia & FLI < 30 |  | 1,889 | 6,825,958 | 0.28 | Reference |  | Reference |  | |
| Prediabetes & FLI < 30 |  | 287 | 902,403 | 0.32 | 1.02 (0.90-1.15) | 0.791 | 0.98 (0.87-1.12) | 0.805 | |
| Normoglycemia & FLI ≥ 30 |  | 1,200 | 1,871,644 | 0.64 | 1.74 (1.61-1.88) | <0.001 | 1.30 (1.18-1.43) | <0.001 | |
| Prediabetes & FLI ≥ 30 |  | 524 | 607,391 | 0.86 | 2.25 (2.03-2.49) | <0.001 | 1.59 (1.41-1.79) | <0.001 | |
| *P*_trend_ |  |  |  |  |  | <0.001 |  | <0.001 | |
| Stroke |  |  |  |  |  |  |  |  | |
| Normoglycemia & FLI < 30 |  | 1,128 | 6,827,791 | 0.17 | Reference |  | Reference |  | |
| Prediabetes & FLI < 30 |  | 195 | 902,563 | 0.22 | 1.14 (0.98-1.33) | 0.085 | 1.10 (0.94-1.28) | 0.229 | |
| Normoglycemia & FLI ≥ 30 |  | 685 | 1,873,079 | 0.37 | 1.69 (1.53-1.88) | <0.001 | 1.27 (1.12-1.44) | <0.001 | |
| Prediabetes & FLI ≥ 30 |  | 250 | 608,187 | 0.41 | 1.77 (1.53-2.05) | <0.001 | 1.24 (1.05-1.46) | 0.011 | |
| *P*_trend_ |  |  |  |  |  | <0.001 |  | <0.001 | |
| Cardiovascular death |  |  |  |  |  |  |  |  | |
| Normoglycemia & FLI < 30 |  | 236 | 6,832,616 | 0.03 | Reference |  | Reference |  | |
| Prediabetes & FLI < 30 |  | 44 | 903,407 | 0.05 | 1.13 (0.82-1.57) | 0.450 | 1.06 (0.77-1.47) | 0.723 | |
| Normoglycemia & FLI ≥ 30 |  | 222 | 1,876,089 | 0.12 | 2.06 (1.69-2.51) | <0.001 | 1.26 (1.00-1.60) | 0.055 | |
| Prediabetes & FLI ≥ 30 |  | 94 | 609,358 | 0.15 | 2.49 (1.94-3.20) | <0.001 | 1.38 (1.03-1.85) | 0.030 | |
| *P*_trend_ |  |  |  |  |  | <0.001 |  | 0.023 | |

Abbreviation: CI, confidence interval; MACE, major adverse cardiovascular events. ^a^Incidence for 1000 person-years. Model 1: Adjusted for age and sex. Model 2: Adjusted for age, sex, income, smoking status, alcohol consumption, regular physical activity, body weight, hypertension, dyslipidemia, and chronic kidney disease.

| **Supplementary Table 7.** Incidence rates and hazard ratios for cardiometabolic outcomes in young adults based on prediabetes and hepatic steatosis status (5-year outcome) | | | | | | | | |  |
| --- | --- | --- | --- | --- | --- | --- | --- | --- | --- |
|  |  | Event | Duration (person-years) | Incidence Rate^a^ | Hazard Ratio (95% CI) | | | | |
|  |  |  |  |  | Model 1 | *P*-value | Model 2 | *P*-value | |
| Incident diabetes |  |  |  |  |  |  |  |  | |
| Normoglycemia & FLI < 60 |  | 1,326 | 3,531,787 | 0.38 | Reference |  | Reference |  | |
| Prediabetes & FLI < 60 |  | 1,091 | 538,649 | 2.03 | 5.21 (4.80-5.65) | <0.001 | 4.60 (4.24-4.99) | <0.001 | |
| Normoglycemia & FLI ≥ 60 |  | 998 | 283,145 | 3.52 | 9.31 (8.52-10.17) | <0.001 | 3.52 (3.17-3.89) | <0.001 | |
| Prediabetes & FLI ≥ 60 |  | 1,506 | 116,140 | 12.97 | 33.05 (30.46-35.85) | <0.001 | 12.05 (10.93-13.29) | <0.001 | |
| *P*_trend_ |  |  |  |  |  | <0.001 |  | <0.001 | |
| Composite MACE |  |  |  |  |  |  |  |  | |
| Normoglycemia & FLI < 60 |  | 902 | 3,532,652 | 0.26 | Reference |  | Reference |  | |
| Prediabetes & FLI < 60 |  | 199 | 540,342 | 0.37 | 1.19 (1.02-1.39) | 0.030 | 1.13 (0.97-1.32) | 0.126 | |
| Normoglycemia & FLI ≥ 60 |  | 207 | 284,345 | 0.73 | 1.99 (1.71-2.33) | <0.001 | 1.45 (1.20-1.75) | <0.001 | |
| Prediabetes & FLI ≥ 60 |  | 113 | 118,872 | 0.95 | 2.43 (1.99-2.97) | <0.001 | 1.69 (1.35-2.12) | <0.001 | |
| *P*_trend_ |  |  |  |  |  |  |  | <0.001 | |
| Myocardial infarction |  |  |  |  |  |  |  |  | |
| Normoglycemia & FLI < 60 |  | 412 | 3,533,415 | 0.12 | Reference |  | Reference |  | |
| Prediabetes & FLI < 60 |  | 90 | 540,492 | 0.17 | 1.20 (0.95-1.51) | 0.127 | 1.15 (0.92-1.45) | 0.223 | |
| Normoglycemia & FLI ≥ 60 |  | 118 | 284,501 | 0.41 | 2.51 (2.03-3.10) | <0.001 | 2.02 (1.56-2.61) | <0.001 | |
| Prediabetes & FLI ≥ 60 |  | 66 | 118,941 | 0.55 | 3.19 (2.44-4.17) | <0.001 | 2.48 (1.82-3.37) | <0.001 | |
| *P*_trend_ |  |  |  |  |  | <0.001 |  | <0.001 | |
| Stroke |  |  |  |  |  |  |  |  | |
| Normoglycemia & FLI < 60 |  | 383 | 3,533,571 | 0.11 | Reference |  | Reference |  | |
| Prediabetes & FLI < 60 |  | 82 | 540,555 | 0.15 | 1.15 (0.90-1.46) | 0.265 | 1.09 (0.86-1.39) | 0.471 | |
| Normoglycemia & FLI ≥ 60 |  | 75 | 284,594 | 0.26 | 1.74 (1.35-2.25) | <0.001 | 1.36 (1.00-1.84) | 0.047 | |
| Prediabetes & FLI ≥ 60 |  | 32 | 118,995 | 0.27 | 1.63 (1.13-2.35) | 0.009 | 1.21 (0.81-1.81) | 0.363 | |
| *P*_trend_ |  |  |  |  |  | <0.001 |  | 0.090 | |
| Cardiovascular death |  |  |  |  |  |  |  |  | |
| Normoglycemia & FLI < 60 |  | 123 | 3,534,340 | 0.03 | Reference |  | Reference |  | |
| Prediabetes & FLI < 60 |  | 31 | 540,710 | 0.06 | 1.27 (0.86-1.89) | 0.232 | 1.14 (0.77-1.70) | 0.505 | |
| Normoglycemia & FLI ≥ 60 |  | 23 | 284,759 | 0.08 | 1.43 (0.91-2.25) | 0.122 | 0.60 (0.35-1.02) | 0.060 | |
| Prediabetes & FLI ≥ 60 |  | 17 | 119,064 | 0.14 | 2.33 (1.39-3.91) | 0.001 | 0.92 (0.51-1.66) | 0.776 | |
| *P*_trend_ |  |  |  |  |  | 0.001 |  | 0.417 | |

Abbreviation: CI, confidence interval; MACE, major adverse cardiovascular events. ^a^Incidence for 1000 person-years. Model 1: Adjusted for age and sex. Model 2: Adjusted for age, sex, income, smoking status, alcohol consumption, regular physical activity, body weight, hypertension, dyslipidemia, and chronic kidney disease.

| **Supplementary Table 8.** Incidence rates and hazard ratios for cardiometabolic outcomes in young adults based on prediabetes and hepatic steatosis status (10-year outcome) | | | | | | | | |  |
| --- | --- | --- | --- | --- | --- | --- | --- | --- | --- |
|  |  | Event | Duration (person-years) | Incidence Rate^a^ | Hazard Ratio (95% CI) | | | | |
|  |  |  |  |  | Model 1 | *P*-value | Model 2 | *P*-value | |
| Incident diabetes |  |  |  |  |  |  |  |  | |
| Normoglycemia & FLI < 60 |  | 6,273 | 6,985,407 | 0.90 | Reference |  | Reference |  | |
| Prediabetes & FLI < 60 |  | 3,933 | 1,058,355 | 3.72 | 3.91 (3.75-4.07) | <0.001 | 3.48 (3.34-3.62) | <0.001 | |
| Normoglycemia & FLI ≥ 60 |  | 4,718 | 550,383 | 8.57 | 8.74 (8.39-9.10) | <0.001 | 3.14 (2.99-3.30) | <0.001 | |
| Prediabetes & FLI ≥ 60 |  | 4,743 | 218,154 | 21.74 | 22.04 (21.15-22.96) | <0.001 | 7.73 (7.36-8.12) | <0.001 | |
| *P*_trend_ |  |  |  |  |  | <0.001 |  | <0.001 | |
| Composite MACE |  |  |  |  |  |  |  |  | |
| Normoglycemia & FLI < 60 |  | 3,259 | 6,993,887 | 0.47 | Reference |  | Reference |  | |
| Prediabetes & FLI < 60 |  | 667 | 1,069,890 | 0.62 | 1.14 (1.05-1.24) | 0.003 | 1.08 (0.99-1.17) | 0.077 | |
| Normoglycemia & FLI ≥ 60 |  | 735 | 562,362 | 1.31 | 2.09 (1.92-2.27) | <0.001 | 1.40 (1.27-1.55) | <0.001 | |
| Prediabetes & FLI ≥ 60 |  | 393 | 234,708 | 1.67 | 2.52 (2.26-2.80) | <0.001 | 1.62 (1.44-1.83) | <0.001 | |
| *P*_trend_ |  |  |  |  |  | <0.001 |  | <0.001 | |
| Myocardial infarction |  |  |  |  |  |  |  |  | |
| Normoglycemia & FLI < 60 |  | 1,813 | 6,998,212 | 0.26 | Reference |  | Reference |  | |
| Prediabetes & FLI < 60 |  | 375 | 1,070,740 | 0.35 | 1.16 (1.04-1.30) | 0.008 | 1.12 (1.00-1.25) | 0.058 | |
| Normoglycemia & FLI ≥ 60 |  | 426 | 563,217 | 0.76 | 2.20 (1.97-2.45) | <0.001 | 1.54 (1.35-1.75) | <0.001 | |
| Prediabetes & FLI ≥ 60 |  | 233 | 235,167 | 0.99 | 2.74 (2.38-3.16) | <0.001 | 1.86 (1.59-2.19) | <0.001 | |
| *P*_trend_ |  |  |  |  |  | <0.001 |  | <0.001 | |
| Stroke |  |  |  |  |  |  |  |  | |
| Normoglycemia & FLI < 60 |  | 1,229 | 6,999,387 | 0.18 | Reference |  | Reference |  | |
| Prediabetes & FLI < 60 |  | 238 | 1,071,022 | 0.22 | 1.07 (0.93-1.23) | 0.328 | 1.01 (0.88-1.16) | 0.866 | |
| Normoglycemia & FLI ≥ 60 |  | 244 | 563,744 | 0.43 | 1.86 (1.62-2.15) | <0.001 | 1.27 (1.07-1.51) | 0.006 | |
| Prediabetes & FLI ≥ 60 |  | 133 | 235,474 | 0.56 | 2.26 (1.88-2.71) | <0.001 | 1.46 (1.19-1.80) | <0.001 | |
| *P*_trend_ |  |  |  |  |  | <0.001 |  | <0.001 | |
| Cardiovascular death |  |  |  |  |  |  |  |  | |
| Normoglycemia & FLI < 60 |  | 286 | 7,003,814 | 0.04 | Reference |  | Reference |  | |
| Prediabetes & FLI < 60 |  | 72 | 1,071,894 | 0.07 | 1.29 (0.99-1.67) | 0.055 | 1.17 (0.90-1.52) | 0.232 | |
| Normoglycemia & FLI ≥ 60 |  | 89 | 564,635 | 0.16 | 2.43 (1.90-3.10) | <0.001 | 1.22 (0.90-1.64) | 0.199 | |
| Prediabetes & FLI ≥ 60 |  | 39 | 235,943 | 0.17 | 2.37 (1.68-3.33) | <0.001 | 1.11 (0.75-1.63) | 0.598 | |
| *P*_trend_ |  |  |  |  |  | <0.001 |  | 0.251 | |

Abbreviation: CI, confidence interval; MACE, major adverse cardiovascular events. ^a^Incidence for 1000 person-years. Model 1: Adjusted for age and sex. Model 2: Adjusted for age, sex, income, smoking status, alcohol consumption, regular physical activity, body weight, hypertension, dyslipidemia, and chronic kidney disease.

| **Supplementary Table 9.** Incidence rates and hazard ratios for liver-related outcomes in young adults based on prediabetes and hepatic steatosis status | | | | | | | | |  |
| --- | --- | --- | --- | --- | --- | --- | --- | --- | --- |
|  |  | Event | Duration (person-years) | Incidence Rate^a^ | Hazard Ratio (95% CI) | | | | |
|  |  |  |  |  | Model 1 | *P*-value | Model 2 | *P*-value | |
| Incident HCC |  |  |  |  |  |  |  |  | |
| Normoglycemia & FLI < 60 |  | 5,493 | 8,030,068 | 0.68 | Reference |  | Reference |  | |
| Prediabetes & FLI < 60 |  | 985 | 1,235,590 | 0.80 | 1.05 (0.98-1.13) | 0.143 | 1.04 (0.97-1.12) | 0.248 | |
| Normoglycemia & FLI ≥ 60 |  | 676 | 650,963 | 1.04 | 1.27 (1.17-1.37) | <0.001 | 1.09 (0.99-1.20) | 0.069 | |
| Prediabetes & FLI ≥ 60 |  | 345 | 271,373 | 1.27 | 1.49 (1.33-1.67) | <0.001 | 1.28 (1.14-1.45) | <0.001 | |
| *P*_trend_ |  |  |  |  |  | <0.001 |  | <0.001 | |
| Liver-related death |  |  |  |  |  |  |  |  | |
| Normoglycemia & FLI < 60 |  | 52 | 8,054,814 | 0.01 | Reference |  | Reference |  | |
| Prediabetes & FLI < 60 |  | 12 | 1,239,845 | 0.01 | 1.23 (0.65-2.31) | 0.529 | 1.19 (0.63-2.25) | 0.587 | |
| Normoglycemia & FLI ≥ 60 |  | 24 | 653,890 | 0.04 | 3.97 (2.39-6.60) | <0.001 | 5.91 (3.11-11.25) | <0.001 | |
| Prediabetes & FLI ≥ 60 |  | 11 | 272,920 | 0.04 | 4.05 (2.07-7.94) | <0.001 | 5.58 (2.55-12.22) | <0.001 | |
| *P*_trend_ |  |  |  |  |  | <0.001 |  | <0.001 | |

Abbreviation: CI, confidence interval; HCC, hepatocellular carcinoma. ^a^Incidence for 1000 person-years. Model 1: Adjusted for age and sex. Model 2: Adjusted for age, sex, income, smoking status, alcohol consumption, regular physical activity, body weight, hypertension, dyslipidemia, and chronic kidney disease.
